# Electrocardiographic and scintigraphic evaluation of patients with subclinical hyperthyroidism during workout

**DOI:** 10.1007/s12020-016-0877-x

**Published:** 2016-02-09

**Authors:** Grzegorz Kaminski, Mirosław Dziuk, Ewelina Szczepanek-Parulska, Ariadna Zybek-Kocik, Marek Ruchala

**Affiliations:** Department of Endocrinology and Isotope Therapy, Military Institute of Medicine, Szaserów St 128, 04-141 Warsaw, Poland; Department of Nuclear Medicine, Military Institute of Medicine, Szaserów St 128, 04-141 Warsaw, Poland; Department of Endocrinology, Metabolism and Internal Medicine, Poznan University of Medical Sciences, 49 Przybyszewskiego St, 60-355 Poznan, Poland

**Keywords:** Subclinical hyperthyroidism, Perfusion scintigraphy, Exercise electrocardiogram, Workout

## Abstract

Subclinical hyperthyroidism (sHT) was found to be associated with elevated heart rate, blood pressure and increased risk of extrasystoles. However, the full clinical relevance of morphological and functional implications of sHT on the cardiovascular system is still a matter of debate. The aim of the study was to prospectively assess the influence of endogenous sHT on exercise capacity and cardiac function during workout with the use of exercise electrocardiography (ExECG) and perfusion scintigraphy. The studied group consisted of 44 consecutively recruited patients diagnosed with sHT. In all patients, ExECG, followed by post-exercise myocardial perfusion imaging, was performed. Both ExECG and scintigraphy were performed twice—in the state of sHT and after euthyroidism was restored. An average time period of exercise test was significantly longer in the state of euthyroidism than in sHT. An average oxygen consumption during exercise test was also higher after euthyroidism was achieved when compared to sHT. The end-diastolic and end-systolic volume indexes, stroke volume index and cardiac index were significantly larger in patients with sHT if compared values achieved after euthyroidism restoration. Stroke volume index was negatively correlated with TSH, and positively with free thyroid hormones values in the state of sHT, before euthyroidism was achieved. Cardiac index was positively correlated with free thyroid hormones levels. The obtained results indicate worse physical capacity in subjects with sHT and improvement of several parameters assessed during ExECG and perfusion scintiscan after therapy. Observed changes might reflect the mechanism of the deleterious effect exerted by sHT on the heart.

## Introduction

Subclinical hyperthyroidism (sHT) is by definition a condition characterized by low or undetectable TSH concentration with free thyroid hormones maintained within the reference ranges [1]. The prevalence of sHT is estimated at about 1 % of general population [2]. It has been demonstrated that sHT exerts negative impact on quality of life and increases the risk of cardiovascular mortality [3–5]; however, data on the influence of sHT on overall mortality are inconsistent [6–9]. On the other hand, reports have demonstrated that the negative impact of sHT on cardiovascular risk, which is recognized in young adults, is less evident in elderly subjects aged 85 years or older [10]. One of the most important parameter modified by sHT is cardiovascular performance. Subclinical thyroid hyperfunction was found to be associated with elevated heart rate and blood pressure as well as increased risk of both supraventricular and ventricular extrasystoles [4, 11, 12]. What is more, sHT might also affect hemodynamics by the negative impact on left ventricular diastolic function and hypertrophy [13]. Although the harmful impact of overt hyperthyroidism on the cardiovascular system is well documented, the clinical relevance of morphological and functional implications of sHT is still a matter of debate [1, 4, 11, 13].

The aim of the study was to prospectively assess the influence of endogenous sHT on exercise capacity and cardiac function during workout with the use of electrocardiographic exercise treadmill test (ExECG) and perfusion scintigraphy.

## Subjects and methods

The studied group consisted of 44 consecutively recruited patients (37 women and 7 men), aged from 22–65 years, mean value 45.9 ± 11.0 diagnosed with sHT. The same cohort of patients had already been studied in the two previous papers of a cycle [11, 13]. The patients were enroled to the study from a group of 1080 subjects referred to our department because of hyperthyroidism (ICD 10—E.05) from April of 2002 to December 2004. Patients were diagnosed with sHT on the basis of the following laboratory criteria:

- decreased thyroid-stimulating hormone (TSH) concentration, below lower normal limit (<0.36 uIU/ml)—at least twice, measured in a period of 6 months;
- normal free thyroid hormones: free triiodothyronine—FT3 (3.5–7.9 pmol/l) and free thyroxin—FT4 (7.64–19.7 pmol/l)—at least twice, measured in a period of 6 months;
- negative anti-thyroid autoantibodies: anti-thyroid peroxidase (TPOAb), anti-thyroglobulin (TgAb) and anti-TSH receptor (TRAb).

A group of patients was selected in whom sHT was due to: toxic multinodular goitre, autonomous nodule or diffused thyroid autonomy. The patients had negative history of other diseases, including cardiovascular and metabolic diseases or were taking no medications (i.e. betablockers). No symptoms of cardiovascular diseases when Holter electrocardiography and echocardiography were performed. Other possible causes of sHT were excluded, i.e. Graves’ disease, thyroiditis, pregnancy, secondary hypothyroidism and euthyroid sick syndrome. After sHT diagnosis, the patients were subjected to radioiodine treatment. The dose of radioisotope was calculated with the use of the following formula [14]:

$$A = \frac{{(100\text{-}150\,\mu {\text{Ci}}) \cdot m}}{{{\text{RAIU}}_{24} }},$$

where *A*—activity (mCi), *m*—mass of hyperthyroid tissue (g) and RAIU_24_—iodine intake after 24 h expressed as decimal.

Authors assessed the volume of the hot or autonomous thyroid nodules (visualized in thyroid scintiscan) in millilitres during the thyroid ultrasound and assumed that mass in grams is approximately equal to the volume in millilitres. The mean dose of given radioiodine was 448.81 ± 209.79 MBq.

The protocol of the study was approved by the local ethical committee and all patients gave written informed consent to participate.

In all patients ExECG, followed by post-exercise, myocardial perfusion imaging was performed. In all patients, both ExECG and scintigraphy were performed twice—in the state of sHT and after restoration of euthyroidism. Subclinical hyperthyroidism at the moment of the test was confirmed to last for at least 6 months (mean duration of about 1 year), while euthyroidism was defined as complete normalization of TSH for at least 6 months. The treadmill test was performed according to Bruce protocol and standards of American Heart Association (AHA) for exercise tests [15, 16]. Patients did not take medications and remained fasting for at least 3 h prior to the test. During the test, blood pressure was controlled in 3-min interval, during second minute of each phase. The period of the test was symptom limited and conducted until patient achieved at least 85 % of maximum heart rate, calculated with the following formula: HR_max_ = 220—age (years). According to AHA, indications for preterm termination of the test appeared in none of the patients [16].

By the means of ExECG, efficiency of the cardiovascular system was assessed. Oxygen consumption was expressed in metabolic equivalents (METS). Moreover, blood pressure, heart rate and double product, defined as systolic blood pressure multiplied by heart rate, were interpreted.

One minute before the end of each exercise test, 740 MBq of isonitryl methoxy-isobutyl (MIBI) labelled with technetium-99 m (99mTc-MIBI) was given intravenously. Forty minutes following the ExECG a gated perfusion scintigraphy (GSPECT) with the use of dual-head gamma camera Varicam, Elscint, was performed. Similar to exercise tests, GSPECT was performed twice in each patient—on diagnosis of sHT and after euthyroidism was achieved. Both MIBI and technetium generator were manufactured by The Research and Development Centre for Isotopes in Świerk, Poland. Continuous method of recording was used. The head of gamma camera was rotated on elliptical orbit from right anterior oblique (RAO-45°) position to left posterior oblique (LPO-45°) position. Thirty images were obtained every 6°. Recording of the activity was carried out for 20 s on each image in a matrix of 64 × 64. During the reconstruction, standard modes of correction (energetics, linearity, sensitivity, centre of rotation and radioactive decay) were adopted. Butterworth rear projection filter was used (order of 5, cut-off of 0.5/cm). No absorption correction was performed. In the case of unintentional movement of the patient, a programme for automatic motion correction was used.

For the analysis of perfusion and dysfunction of the left ventricle, XpertPro system was used with CEQUAL (Emory Cedars Quantitative Analysis) software by Elscint and data development station Quantitative Gated Spect (QGS) software by Hermes Nuclear Diagnostics. Images of the heart have been reconstructed in three planes orthogonal to the axis of the heart: the vertical long axis, horizontal long axis and short axis.

Determination of the degree of perfusion disturbances in particular segments of the left ventricle wall was performed in a semi-quantitative way using a scale, where 0—none, 1—slight, 2—moderate and 3—considerable. None of the subjects presented the indexes of impaired systolic perfusion of the left ventricle.

Systolic and diastolic function of the left ventricle was assessed by gating the RR interval for 8 sub-periods. For evaluation of post-exercise systolic left ventricle function, the following parameters were used:

- End-diastolic volume—EDV (ml);
- End-systolic volume—ESV (ml);
- Ejection fraction of the left ventricle—EF (%);
- PER (Peak Ejection Rate)—maximum left ventricular emptying calculated as the greatest percentage change in volume that occurs in one second from the time when EDV (EDV%/s) is achieved;
- TPER (Time to Peak Ejection Rate)—time from maximum diastole to the point of PER (s) and
- TEDES (Time from End-diastole to End-systole)—time from achievement of EDV to the achievement of ESV (s).

**Fig. 1:**
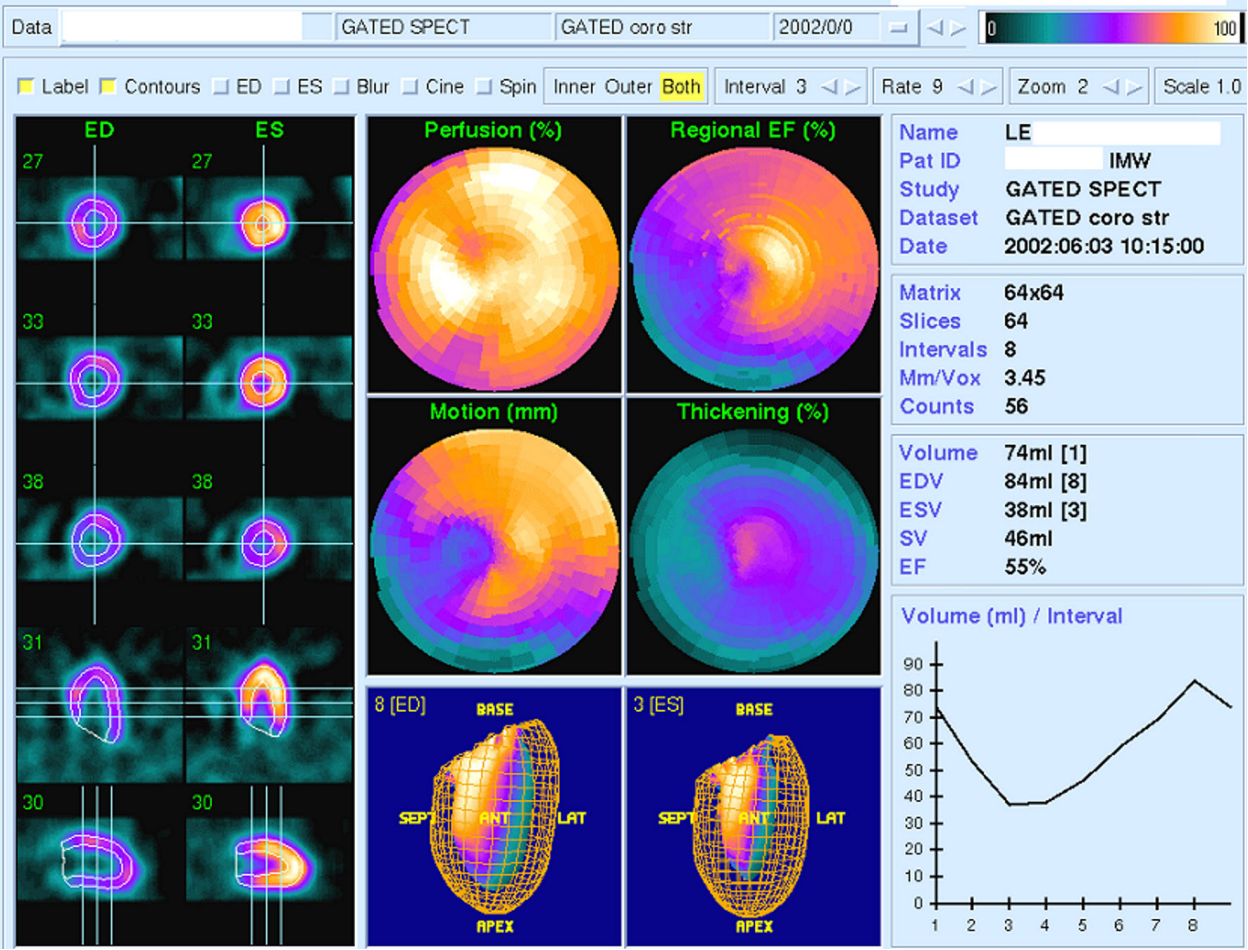
A sample test results of the ECG-gated perfusion scintigraphy performed before the radioiodine therapy that shows no cardiological problems. Acquisition was carried out in 40 min after injection of 740 MBq of Tc-99 m MIBI on top of the stress test carried out on a treadmill. The column on the left depicts cross sections through the myocardium in each sub-period of the contraction of the left ventricle. ED (end-diastolic) means the period of maximum diastole of the left ventricle, ES (end-systolic) means the period of maximum contraction of the left ventricle. The top three rows in this column sections depict the short axis of the heart. The following sections represent the horizontal long axis and vertical long axis of the heart. In the middle column, there are successively from the top: polar map of perfusion of the left ventricle (Perfusion) as a percentage of the maximum uptake, polar map of wall motion (motion) in millimetres, and a three-dimensional image of the left ventricular cavity during the end-diastole. In the column on the right there are: map of regional left ventricular ejection fraction (EF Regional), polar map of the thickening during systole and three-dimensional image of the left ventricle during end-systolic period. In addition, the image presented personal data and acquisition parameters. In the lower right corner, there is a curve depicting the volume changes in the sub-periods of heart contraction. *Sep* septum, *LAT* lateral wall

In the evaluation systolic function and perfusion of the left ventricle, the final report was included (Fig. 1):

- sectional views through the heart in the short axis (SA), horizontal long axis (HLA) and vertical long axis (VLA) at rest and in the phase of the maximum contraction of the left ventricle;
- perfusion polar maps (Perfusion), regional ejection fraction (EF Regional), increase in thickness (Thickening) and motion (Motion), of the walls of the left ventricle along with the colour scale representing the degree of perfusion;
- a three-dimensional image of the left ventricle systole and diastole maximum at an optional projection;
- a chart showing the number of counts from the left ventricle during each time sub-period;
- automatically calculated ejection fraction (EF);
- automatically calculated stroke volume (SV);
- end-diastolic volume (EDV) and
- end-systolic volume (ESV).

The values of indices of left ventricular volume: EDVI, ESVI and SVI calculated by the following formulas: EDVI = EDV/BSA, ESVI = ESV/BSA and SVI = SV/BSA (ml/m^2^).

The diastolic function of the left ventricle was evaluated using the following parameters:

- PFR (Peak Filling Rate)—the peak filling velocity ratio. The PFR referred to the end-systolic volume and presented as the highest percentage increase of the volume in one second [ESV%/s] from the time when ESV was achieved and
- TPFR (Time to Peak Filling Rate)—the time period from the moment when the ESV was achieved to the moment of PFR.

The complete evaluation of patients was performed twice—at the moment of sHT diagnosis confirmation and 6 months after transition to euthyroidism, confirmed by complete TSH normalization.

The difference between particular parameters assessed during exercise test in the state of sHT and euthyroidism was analysed with the use of T-Student test for dependent variables. The correlations between the parameters of exercise test or perfusion scintigraphy and hormonal tests results (both in sHT and euthyroid state) were calculated with the use of Pearson’s test. The difference between particular parameters assessed during perfusion scintigraphy before and following the therapy for subclinical hyperthyroidism was checked with T-Student test for dependent variables. Normality was analysed by Shapiro–Wilk test, and the equality of variances was assessed with the use of Levene’s test. The data were analysed statistically with the use of STATISTICA software by StatSoft.

## Results

### Thyroid function status

The results of hormonal tests were as follows (in sHT state and after restoration of euthyroidism, respectively): TSH 0.16 ± 0.10 mIU/L versus 1.32 ± 0.75 (*p* < 0.005), FT4 14.16 ± 2.37 pmol/L versus 13.05 ± 1.85 and FT3 6.48 ± 0.69 pmol/L versus 5.77 ± 0.57. The interval between all performed examinations was on average 12.5 months.

### Exercise test

A mean heart rate in the state of subclinical hyperthyroidism and after restoration of euthyroidism was as follows: 78.4 ± 6.8 versus 76.0 ± 8.0 beats/min, *p* = 0.004). No patients presented important arrhythmia during the test. An average time period of exercise test was significantly longer in the state of euthyroidism than in sHT (8.21 vs. 7.17 min, *t* = −3.5 *p* = 0.001). An average oxygen consumption during exercise test expressed in METS was higher after euthyroidism was achieved if compared to sHT (10.73 vs. 9.27, *t* = −4.2 *p* < 0.00001), while maximum double product was higher in sHT (27,870) and decreased to 26,327 after euthyroidism was restored (*t* = 2.5 *p* = 0.018). The parameters measured during ExECG are presented in Table 1. There was a significant negative correlation between METS and FT3/FT4 coefficient before therapy (*r* = −0.348, *p* = 0.041) as well as a significant positive correlation between maximum double product and FT3/FT4 coefficient (*r* = 0.329, *p* = 0.041).

**Table 1:** Values of the parameters measured during exercise ECG (ExECG) in sHT and after euthyroidism was restored

Parameter	sHT				Euthyroidism				*p*
	Mean	Confidence interval		SD	Mean	Confidence interval		SD	
		−95 %	+95 %			−95 %	+95 %		
Exercise duration (min)	7.17	6.33	8.01	2.76	8.21	7.29	9.13	3.02	**0.001**
METS	9.27	8.349	10.18	3.016	10.73	9.674	11.78	3.458	**<0.001**
RR-max (mmHg)	171.36	164.79	177.94	21.630	166.48	160.68	172.27	19.065	0.139
Maximum double product (b*mmHg/min)	27,870.1	26,759.8	28,980.4	3652.1	26,531.1	25,462.0	27,600.3	3516.5	**0.018**
HRmax (b/min)	162.82	157.35	168.29	18.000	164.98	159.24	170.72	18.882	0.334
percentage of estimated HRmax (%)	93.09	90.13	96.05	9.728	95.41	92.86	97.95	8.370	0.086

Bold values indicate statistical significance (*p* < 0.05)

*sHT* subclinical hypothyroidism, *SD* standard deviation, *METS* metabolic equivalents, *RR-max* maximum blood pressure, *HRmax* maximum heart rate

### Perfusion scintigraphy

The end-diastolic volume index (EDVI) was significantly larger (45.82 ml/m^2^) in patients with sHT if compared to the value 40.62 ml/m^2^ obtained after euthyroid state was achieved (*t* = 3.2 *p* = 0.002). Similar observation concerned end-systolic volume index (ESVI), which was 19.45 ml/m^2^ and dropped to 16.40 ml/m^2^ with the therapy (*t* = 2.3 *p* = 0.025). Two more parameters were also found to be higher in patients with sHT if compared to those measured after restoration of euthyroidism: stroke volume index (SVI, 26.36 vs. 24.21 ml/m^2^, respectively, *t* = 1.9, *p* = 0.042) and cardiac index (CI 2.35 vs. 2.14 ml/m^2^, respectively, *t* = 2.0, *p* = 0.041). The parameters measured during perfusion scintigraphy are presented in Table 2.

**Table 2:** The results of the parameters measured during perfusion scintigraphy in sHT and after euthyroidism were restored

Parameter	sHT				Euthyroidism				p
	Mean	Confidence interval		SD	Mean	Confidence interval		SD	
		−95 %	+95 %			−95 %	+95 %		
Post-exercise EF	62.35	59.99	64.71	7.67	63.77	61.35	66.19	7.87	0.102
PFR (%/s)	336.00	297.49	374.51	110.37	319.71	279.46	359.95	115.35	0.427
PER (%/s)	359.56	317.19	401.93	121.43	373.32	341.08	405.57	92.42	0.432
TPFR (ms)	517.32	477.79	556.86	113.31	545.82	510.09	581.55	102.40	0.183
TPER (ms)	198.97	160.90	237.04	109.11	176.74	150.74	202.73	74.51	0.389
TEDES (ms)	365.85	332.81	398.89	94.70	373.56	345.82	401.29	79.49	0.715
EDVI (ml/m^2^)	45.82	42.60	49.04	10.58	40.62	36.23	45.00	14.42	**0.002**
ESVI (ml/m^2^)	19.45	16.44	22.47	9.91	16.40	14.08	18.73	7.65	**0.025**
SVI (ml/m^2^)	26.36	23.21	29.52	10.38	24.21	20.80	27.62	11.22	**0.042**
CI (l/min m^2^)	2.35	2.06	2.63	0.95	2.14	1.83	2.45	1.05	**0.041**

Bold values indicate statistical significance (*p* < 0.05)

*sHT* subclinical hypothyroidism, *SD* standard deviation, *EF* ejection fraction, *PFR* peak filling rate, *PER* peak ejection rate, *TPFR* time to peak filling rate, *TPER* time to peak ejection rate, *TEDES* time from end-diastole to end-systole, *EDVI* end-diastolic volume index, *ESVI* end-systolic volume index, *SVI* stroke volume index, *CI* cardiac index

Moreover, the correlation between particular parameters measured during perfusion scintigraphy and hormonal tests results was assessed. Significant negative correlation was found in sHT between SVI and TSH in patients before therapy (*r* = −0.318, *p* = 0.041), while significant positive correlation was found between SVI and FT3 (*r* = 0.347, *p* = 0.008) as well as SVI and FT4 (*r* = 0.395, *p* = 0.0005). A significant positive correlation before therapy was found also for CI and FT3 (*r* = 0.323, *p* = 0.032) as well as for CI and FT4 (*r* = 0.300, *p* = 0.048).

## Discussion

The presented study constitutes the third one of a cycle in which we demonstrate a profound influence of sHT on cardiovascular performance by prospective assessment of patients with endogenous sHT done before therapy and after euthyroidism was restored, using different methods of diagnosis [11, 13]. In our previous studies, we analysed the results of echocardiography, 24-h ambulatory blood pressure monitoring and 24-h electrocardiography. It was demonstrated that endogenous sHT was associated with statistically significant increase in QT dispersion, incidence of ventricular extrasystoles, elevated nocturnal arterial blood pressure and changes in heart rate variability [11]. Moreover, sHT was associated with increased volume of heart chambers and ascending aorta, increased left ventricle mass and disturbed left ventricle relaxation. The changes were reversible with restoring biochemical euthyroidism [13]. This time, patients with sHT were subjected to physical exercise, and cardiovascular performance was evaluated with exercise ECG and perfusion scintigraphy.

The use of exercise testing in cardiological diagnostics is based on the assumption that exercise may provoke a disclosure of some latent abnormalities in cardiovascular system, which are undetected in examinations performed without load. In healthy people, during physical activity, several parameters including total oxygen consumption, minute volume and heart rate increase to the limit of physical capacity, defined as the maximum effort. Above this threshold, further work is done at the expense of anaerobic processes, and does not generate increase in minute volume and heart rate [17]. For patients, exercise could be limited by the onset of clinical symptoms and electrocardiographic abnormalities. ECG testing generally is recommended for risk assessment in patients with ischemic cardiac disease [18]. The occurrence of ST-segment depression at a reduced workload or persisting into recovery coupled with exertional symptoms is described to be associated with a high risk of cardiovascular events and mortality. In our study, ExECG was used to assess functional capacity and to exclude coronary artery disease in patients with sHT. Similarly, exercise SPECT has also been shown to effectively assess risk of subsequent events in patients with ischemic heart disease, with a low annualized event rate of 1.6 % observed in patients with a normal adenosine SPECT versus 10.6 % in patients with a severely abnormal study (summed stress score more than 13).

It is assumed that an exercise at the level of 85–90 % of maximum effort, expressed as the maximum heart rate limit, is an adequate test to assess blood supply to the heart muscle [16, 19]. No signs of ischaemia at this load reflect good state of coronary circulation. In our study, an average heart rate limit of the patients was 93 % before and 95 % after recovering from sHT. None of the subjects demonstrated neither clinical nor electrocardiographic characteristics of coronary artery disease.

Biondi et al. compared the results of ExECG performed on a bicycle ergometer in 10 patients suffering from exogenous sHT with the results of appropriately matched group of 10 healthy subjects [20]. The findings were consistent with the results of our study.

In another study, 19 patients with exogenous sHT underwent spirometric maximal exercise test. When the results were compared with those obtained in an equally large control group of healthy subjects, it turned out that patient with sHT had significantly reduced exercise capacity, oxygen consumption and anaerobic threshold [21].

The metabolic equivalent (MET) is a unit of oxygen uptake by the human body at rest. It is assumed that 1 MET reflects an absorption of 3.5 ml O_2_ per 1 kg body per minute. A maximum oxygen uptake is influenced by age, gender, habitual physical activity, genetic factors and finally the state of the cardiovascular system. A study conducted at the Mayo Clinic demonstrated that the ability to perform work expressed in metabolic equivalents (METS) was the only variable if an effort on a treadmill is concerned, which was related to total mortality in the studied group [22]. Maximal values of oxygen uptake (VO_2_max) are usually achieved between 15 and 30 years of age and decreases by an average of 8–10 % per decade, both in people leading a sedentary lifestyle, as well as in athletes [23]. It should be noted that despite the fact that at the time of performing repeated tests, the patients were on average 1 year older (VO_2_max theoretically should have decreased during this period by about 1 %), the recovery from sHT resulted in a statistically significant increase in METS by 16 % (from 9.27 to 10.73).

The so-called double product, which is achieved by multiplying the maximum heart rate and systolic blood pressure, allows indirect evaluation of load and consumption of oxygen by the myocardium [24, 25]. ExECG studies conducted in the phase of sHT demonstrated a significantly greater value of the double product and at the same time lower overall oxygen consumption (METS) as well as shortening of exercise duration. This indicates that endogenous sHT is characterized by a decrease in general physical fitness and workout for patient with sHT causes disproportionate burden to the heart. Similarly, in the work by Biondi et al. performed on patients with exogenous sHT, a limited exercise capacity in terms of reduced ability to do the workout (expressed in Watts) and shortening of the effort time were found to be associated with sHT [26]. These authors also found that the group of patients with sHT double product was significantly higher during the same load as compared to a control group of healthy people.

Correlations found in our work between the ExECG parameters and indicators of sHT activity may indicate that a reduction in overall physical capacity and increase in the burden on the heart in the course of endogenous sHT might be due to disturbed ratio between the concentrations of biologically more active triiodothyronine and less active thyroxine (FT3/FT4).

ECG-Gated Single Photon Emission Computed Tomography (GSPECT) is currently a valuable and widely used technique, which allows for assessment of perfusion and function of the left ventricle [27, 28]. In the study, GSPECT was used in order to assess the hemodynamic indices and to exclude coronary artery disease in the studied group of patients with sHT. In a semi-quantitative assessment during GSPECT, none of the patients, either before or after recovering from sHT, demonstrated impairment of myocardial perfusion during contraction, which confirmed earlier exclusion of coronary artery disease. In quantitative evaluation during GSPECT, statistically significant differences between designated parameters, assessed during and after recovering from sHT, were noted.

Our paper is the first in the world literature to evaluate the results of GSPECT examination conducted in patients with endogenous sHT. However, there were two studies in patients with exogenous sHT, in which for the evaluation of cardiac function, a labelled radioisotope was used. Biondi et al. performed a study with a control group, in which a radionuclide angioscintigraphy was performed at rest and during submaximal exercise test in 10 patients with sHT [20]. It was concluded that sHT was associated with a slight non-significant increase in ejection fraction, end-diastolic volume and peak ejection rate as well as statistically significant decrease in peak filling rate.

In another paper, 11 patients with exogenous sHT demonstrated a higher heart rate and not significantly increased ejection fraction [29]. Faber et al. compared the hemodynamic measurements performed with the use of impedance cardiography in a group of 6 women before and after recovery from endogenous sHT following radioiodine therapy [30]. After restoration of euthyroidism, 19 % reduction (*p* < 0.05) in cardiac output and cardiac index as well as 30 % increase in systemic vascular resistance, were observed. In this small group of patients with sHT, no correlation was found between concentrations of hormones and haemodynamic parameters. However, after increasing the sample size, the negative correlation between TSH and the minute cardiac output and an index of aortic compliance as well as positive correlation between TSH and systemic vascular resistance were noted. Moreover, a positive correlation was found between FT3 and a minute cardiac output and an index of aortic compliance as well as negative correlation between FT3 and mean arterial pressure and systemic vascular resistance. In our study, no significant differences in parameters describing left ventricle filling and emptying before and after therapy were noted. It has been shown, however, that this group of patients is characterized by the increase in both end-diastolic and end-systolic volume. Larger difference between these parameters (EDV—ESV) during sHT results in increased stroke volume and cardiac index. Correlations suggest an influence of relative excess concentration of thyroid hormones (also expressed only as TSH suppression) on stroke volume and cardiac index value.

To conclude, the paper constitutes, the first, to prospectively assess the workout performance, and the influence of sHT on the cardiovascular system exercise tolerance in patients with endogenous sHT and after euthyroidism was restored. The obtained results indicate worse physical capacity in subjects with sHT and improvement of several parameters assessed during exercise ECG and perfusion scintiscan after therapy. Observed changes might reflect the mechanism of the deleterious effect exerted by sHT on the heart.
